# Pseudo loss of resistance in epidural space localization

**DOI:** 10.4103/1658-354X.65126

**Published:** 2010

**Authors:** Babita Gupta, Sarita Sharma, Nita D‘souza, Manpreet Kaur

**Affiliations:** *Department of Anesthesia, All India Institute of Medical Sciences, JPNA Trauma Centre, New Delhi, India*

Sir,

Loss of resistance (LOR) is the most commonly used technique to locate the epidural space.[1] LOR to air or saline is often used and is accurate in identifying epidural space in most of the patients. False positive or pseudo-LOR can occur if the needle enters paraspinous muscles or a small cyst in the ligamentum flavum or the interspinous ligaments.[2] However, the exact incidence of false positive LOR is not reported in the literature. We encountered a false positive LOR in a patient with subcutaneous emphysema secondary to chest trauma.

**Figure 1:**
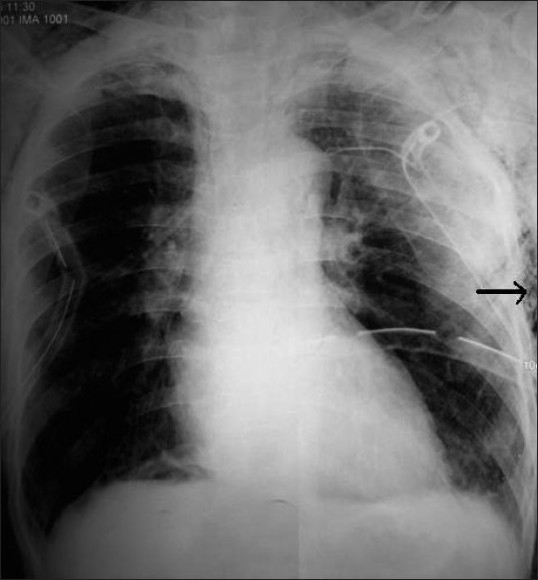
X-ray chest with bilateral intercostal drains; the arrows depicting areas of subcutaneous emphysema

A 56-year-old male patient weighing 50 kg with blunt trauma chest having multiple rib fractures was referred to us for thoracic epidural analgesia. The patient initially had right-sided pneumothorax and subsequently developed left-sided hemo-pneumothorax for which intercostal drains were inserted bilaterally. He had bilateral subcutaneous emphysema all over the chest, upper back, supraclavicular fossa and neck [Figure 1]. The patient presented with tachypnoea, paradoxical respiration and the air entry was decreased on the right side. The visual analogue score (VAS) was 9. After securing intravenous access, the patient was positioned for epidural catheter placement in T8–T9 interspace in sitting position with 18G Touhy needle by midline approach. LOR was elicited at 3.5 cm with air as well as with saline. The epidural catheter, however, could not be advanced beyond 4.5 cm. A second attempt was made in T9–T10 interspace and LOR was obtained at 3.5 cm; again the epidural catheter could not be threaded beyond 4.5 cm. Another attempt was made in T7–T8 interspace and this time LOR was achieved at 4.5 cm. The catheter could be negotiated and was fixed at 9.0 cm mark at the skin level. The patient was shifted to the ward with an epidural infusion of 0.125% bupivacaine and fentanyl 2 mcg/mL, at a rate of 5 mL/h after the test dose and VAS noted was 3.

Epidural space is usually localized beyond 3.0 cm in adults.[3] In our case we got false positive LOR at 3.5 cm. This pseudo-LOR has been described as occurring with the needle entry into the paraspinous muscles or into the cysts that occur when the interspinous ligament degenerates in the lumbar region. Existence of such cysts in the thoracic region of the interspinous ligament is unknown.[4] In the first two attempts, the LOR could have been because of some air pocket in the traversing path of the Touhy needle. The subcutaneous emphysema could have penetrated the deeper planes giving rise to the air pocket. Though such air pockets do not have negative pressure, they can accommodate some air or saline as a result of the pressure exerted on the plunger of the syringe. This air pocket will not allow catheter to pass through. Radiological imaging guidance is helpful in such a scenario but is cumbersome. Various other techniques of epidural space localization have been described such as negative pressure techniques (hanging drop sign, capillary tube method, manometer techniques), loss of resistance methods (syringe loaded syringe, balloon technique), nerve stimulation, and recently, ultrasound guidance.[3,5] However these are limited by equipment availability. Pseudo loss of resistance may be elicited in patients with post-traumatic emphysema due to tracking of air into deeper planes attributable to the impact of injury.
